# Supplementary material for: Improving support for family caregivers: A mixed-methods effect evaluation of an organizational intervention
Source: Palliat Support Care. 2026 Jan 29;24:e39. doi: 10.1017/S1478951525101582 (PMC13166565; doi:10.1017/S1478951525101582)
Supplement: Hoffstädt et al. supplementary material 1 — Hoffstädt et al. supplementary material [file S1478951525101582sup001.docx]

**Action plan template: Support for Family Caregivers intervention**

Healthcare organization:

|  |
| --- |

Department / team:

|  |
| --- |

Project ambassador:

|  |
| --- |

Start of project (date):

|  |
| --- |

**How are family caregivers currently being supported?**

*The main conclusions from the family caregiver journey-workshop regarding current practices in supporting family caregivers.*

What is the team currently satisfied with regarding the support provided to family caregivers?

|  |
| --- |

Which important needs of (bereaved) family caregivers are currently being sufficiently met?

|  |
| --- |

**What would the team like to achieve with the intervention?**

*The main conclusions from the family caregiver journey workshop on what the team would like to achieve.*

Which needs of (bereaved) family caregivers that are currently not sufficiently met does the team to intend to focus on?

|  |
| --- |

Which goals would the team like to achieve? Formulate this SMART (Specific, Measurable, Acceptable, Realistic, Time-Bound) and concise.

|  |
| --- |

**Detailed action plan**

**Goal 1:**

|  |
| --- |

Required materials:

|  |
| --- |

Timeline for achieving the goal:

| From: To: |
| --- |

Actions:

| **What** | **Who** | **When** |
| --- | --- | --- |
|  |  |  |
|  |  |  |
|  |  |  |
|  |  |  |
|  |  |  |

**Goal 2:**

|  |
| --- |

Required materials:

|  |
| --- |

Timeline for achieving the goal:

| From: To: |
| --- |

Actions:

| **What** | **Who** | **When** |
| --- | --- | --- |
|  |  |  |
|  |  |  |
|  |  |  |
|  |  |  |
|  |  |  |

**Goal 3:**

|  |
| --- |

Required materials:

|  |
| --- |

Timeline for achieving the goal:

| From: To: |
| --- |

Actions:

| **What** | **Who** | **When** |
| --- | --- | --- |
|  |  |  |
|  |  |  |
|  |  |  |
|  |  |  |
|  |  |  |

**Goal 4:**

|  |
| --- |

Required materials:

|  |
| --- |

Timeline for achieving the goal:

| From: To: |
| --- |

Actions:

| **What** | **Who** | **When** |
| --- | --- | --- |
|  |  |  |
|  |  |  |
|  |  |  |
|  |  |  |
|  |  |  |

**Goal 5:**

|  |
| --- |

Required materials:

|  |
| --- |

Timeline for achieving the goal:

| From: To: |
| --- |

Actions:

| **What** | **Who** | **When** |
| --- | --- | --- |
|  |  |  |
|  |  |  |
|  |  |  |
|  |  |  |
|  |  |  |

**Step 3: Preparing for implementation**

What are the main barriers among team members and/or within the department or organization to support (bereaved) family caregivers in a structural manner and/or to achieving the goals described above?

|  |
| --- |

What is needed to lower these barriers for individual team members, the team, or the organization?

|  |
| --- |
