# Supplementary material for: Improving support for family caregivers: A mixed-methods effect evaluation of an organizational intervention
Source: Palliat Support Care. 2026 Jan 29;24:e39. doi: 10.1017/S1478951525101582 (PMC13166565; doi:10.1017/S1478951525101582)
Supplement: Hoffstädt et al. supplementary material 2 — Hoffstädt et al. supplementary material [file S1478951525101582sup002.docx]

**Table 1.** Healthcare professionals’ reported care they provided before and after the patient’s death (N=97)

| **During the illness trajectory** |  |  | |  |  |
| --- | --- | --- | --- | --- | --- |
| *Based on the previous 6 months* | % or  mean [SD] | n | | % or  mean [SD] | n |
| In conversations with family caregivers, do you ask how they are doing and/or if there is anything you can do for them?  Yes  No | 98%  2% | 94  2 | | 100%  0% | 96  0 |
| If yes on the question above, how many family caregivers out of 10 do you ask this? | 8 [3] | | 90 | 8 [2] | 92 |
| In conversations with family caregivers, do you ask what is important to them?  Yes  No | 79%  21% | 73  19 | | 93%  7% | 85  6 |
| If yes on the question above, how many family caregivers out of 10 do you ask this? | 8 [3] | 71 | | 7 [3] | 82 |
| *Based on a recent case* |  | | |  | |
| Did you have any contact with the family caregiver(s)?  Yes, more than once  Yes, once  No | 77%  18%  5% | 67  16  4 | | 92%  8%  0% | 69  6  0 |
| In conversations with the family caregiver(s), did you ask how they were doing and/or if there was anything you could do for them?  Yes  No | 99%  1% | 80  1 | | 99%  1% | 72  1 |
| Did you give the family caregiver(s) information about specific facilities and support options for themselves (for example, about home care or using volunteers)?  Yes  No | 51%  49% | 41  39 | | 65%  35% | 47  25 |
| Did you give the family caregiver(s) information about the patient’s disease, such as its course, appropriate care, and treatment options?  Yes  No | 74%  26% | 56  20 | | 84%  16% | 54  10 |
| Did you give the family caregiver(s) the opportunity to ask questions about the patient’s disease(s) and the medical treatment and care?  Yes  No | 84%  16% | 62  12 | | 95%  5% | 53  3 |
| Did you ask the family caregiver(s) if they needed additional (professional) support for themselves, at or outside of the care facility?  Yes  No | 49%  51% | 36  38 | | 60%  40% | 35  23 |
| Did you refer the family caregiver(s) for additional (professional) support?  Yes  No | 30%  70% | 22  51 | | 37%  63% | 21  36 |
| Did you make agreements with the family caregiver(s) about their role as a co-caregiver? That is, about what care would be performed by the family caregiver(s), and what care was taken over by healthcare professionals?  Yes  No | 60%  40% | 43  29 | | 79%  21% | 44  12 |
| *Based on the previous 3 months* | | | | | |
| Overall, how would you evaluate the attention you have for family caregivers’ before the patient’s death?  Excellent  Very good  Good  Mediocre  Poor | 2%  26%  56%  14%  1% | 2  22  47  12  1 | | 4%  34%  61%  2%  0% | 3  29  52  2  0 |
| **After the patient’s death** |  |  | |  |  |
| *Based on a recent case* |  |  | |  |  |
| Did you have any contact with the family caregiver(s) after the death?  Yes, more than once  Yes, once  No | 21%  29%  51% | 17  24  42 | | 29%  34%  37% | 21  25  27 |
| Did you contact the family caregiver(s) by telephone after the patient’s death?  Yes  No | 71%  29% | 29  12 | | 78%  23% | 31  9 |
| Did you invite the family caregiver(s) to a follow-up conversation in the weeks after death?  Yes  No | 61%  39% | 25  16 | | 60%  40% | 24  16 |
| Did you give information about the grieving process and the options for additional (professional) support outside your care facility?  Yes  No | 51%  49% | 20  19 | | 53%  48% | 21  19 |
| Did you refer the family caregiver(s) for additional support?  Yes  No | 26%  74% | 10  28 | | 18%  82% | 6  28 |
| *Based on the previous 3 months* | | | | | |
| Overall, how would you evaluate the care you provide to family caregivers after the patient’s death?  Excellent  Very good  Good  Mediocre  Poor | 1%  9%  54%  32%  5% | 1  7  44  26  4 | | 4%  19%  61%  11%  5% | 3  16  51  9  4 |

**Table 2.** Family caregivers’ reported care they received before and after the patient’s death

|  | Pre (N = 123) | | Post (N = 99) | | | |
| --- | --- | --- | --- | --- | --- | --- |
|  | % n % n | | | | | |
| **During the illness trajectory** |  | |  | | | |
| Did healthcare professionals ask you how you were doing and/or if there was anything they could do for you?  Yes  No  I don’t know | 74%  19%  7% | 91  23  9 | | 87%  11%  2% | | 85  11  2 |
| Did healthcare professionals ask you what is important to you?  Yes  No  I don’t know | 62%  28%  11% | 75  34  13 | | 65%  21%  13% | | 64  21  13 |
| Did healthcare professionals give you information about specific facilities and support options for you, such as the use of home care, or volunteers?  Yes  No  I don’t know | 65%  27%  8% | 78  32  10 | | 64%  24%  12% | | 62  23  12 |
| Did healthcare professionals give you general information about the healthcare organization, such as information about your contact person and any visiting hours?  Yes  No  I don’t know | 82%  9%  9% | 101  11  11 | | 85%  12%  3% | | 82  11  3 |
| Did healthcare professionals give you information about your relative’s disease, such as its course, appropriate care, and treatment options?  Yes  No  I don’t know | 84%  11%  5% | 102  13  6 | | | 77%  18%  5% | 74  17  5 |
| Did healthcare professionals give you the opportunity to ask questions about your relative’s disease and the medical treatment and care?  Yes  No  I don’t know | 94%  6%  0% | 116  7  0 | | | 94%  4%  2% | 89  4  2 |
| Did healthcare professionals ask you if you needed additional support for yourself (support from a volunteer or professional)?  Yes  No  I don’t know | 47%  39%  14% | 57  48  17 | | | 46%  42%  13% | 44  40  12 |
| Did healthcare professionals refer you for additional support, if you needed it (support from a volunteer or professional)?  Yes  No  I did not need this | 24%  30%  46% | 30  37  56 | | | 21%  28%  51% | 20  27  49 |
| Did healthcare professionals make agreements with you about your role in providing care together with healthcare professionals? That is, about what care would be performed by the family caregivers, and what care was taken over by the healthcare professionals?  Yes  No, but I did not need this  No, but I would have appreciated it | 61%  32%  7% | 74  39  9 | | | 50%  42%  9% | 47  39  8 |
| Do you feel that you received sufficient guidance and support (for example, in dealing with the approaching end, with life questions, practical problems, etc.)?  Yes  Partially  No  I don’t know | 64%  22%  11%  4% | 77  26  13  5 | | | 68%  14%  13%  5% | 65  13  12  5 |
| Do you feel that healthcare professionals paid sufficient (real) attention to you as a person (attention for who you are, with your own personality, beliefs, way of life, etc.)?  Yes  Partially  No  I don’t know | 61%  21%  11%  7% | 75  26  14  8 | | | 74%  14%  7%  5% | 71  13  7  5 |
| In the last weeks before your relative’s death, did you have any bad experience with the care provided to you by the healthcare professionals?  Yes  No | 15%  85% | 18  104 | | | 9%  91% | 9  89 |
| **In the last week of the patient’s life** |  |  | | |  |  |
| How often, in the last week of life of your relative, did the healthcare providers offer the kind of emotional support you wanted?  Always  Most of the time  Sometimes  Never  I did not want emotional support / did not need it | 24%  24%  19%  10%  24% | 29  29  23  12  29 | | | 31%  24%  23%  2%  20% | 30  23  22  2  19 |
| How often, in the last week of life of your relative, did the healthcare providers offer the kind of support with existential questions you wanted?  Always  Most of the time  Sometimes  Never  I did not want support with existential questions / did not need it | 13%  9%  15%  17%  45% | 16  11  18  20  54 | | | 9%  8%  13%  10%  59% | 9  8  12  10  57 |
| How often, in the last week of life of your relative, did the healthcare providers offer the kind of practical support you wanted?  Always  Most of the time  Sometimes  Never  I did not want practical support / did not need it | 28%  15%  18%  13%  27% | 33  18  22  15  32 | | | 17%  16%  15%  12%  40% | 16  15  14  11  37 |
| Overall, how would you evaluate the care that you yourself received from healthcare professionals during the last week of your relative’s life?  Excellent  Very good  Good  Mediocre  Poor  Healthcare providers did not provide care to me | 22%  30%  28%  8%  0%  13% | 26  36  33  9  0  16 | | | 41%  25%  18%  4%  1%  12% | 39  24  17  4  1  11 |
| **After the patient’s death** |  |  | | |  |  |
| In the weeks following your relative’s death, were you contacted by any healthcare professional from the organization where your relative spent the most time in their last week, or from whom your relative received care?  Yes  No | 63%  37% | 76  44 | | | 73%  27% | 70  26 |
| Were you invited to a follow-up conversation (by telephone) after the death of your relative?  Yes  No, and I did not need this  No, but I felt a need for this | 39%  46%  15% | 48  56  19 | | | 59%  37%  4% | 56  35  4 |
| Have you had a follow-up conversation?  Yes  No, and I did not need this  No, but I felt a need for this | 33%  54%  13% | 40  65  16 | | | 46%  46%  9% | 43  43  8 |
| Did a healthcare professional from the organization where your relative spent the most time in their last week, or from whom your relative received care, give you information about the grieving process and options for additional support?  Yes  No | 38%  62% | 45  73 | | | 37%  63% | 33  57 |
| Overall, how would you evaluate the care that you received from all involved healthcare professionals after your relative’s death?  Excellent  Very good  Good  Mediocre  Poor  Healthcare providers did not provide care to me after my relative’s death | 15%  24%  20%  11%  2%  29% | 18  29  25  13  3  35 | | | 32%  21%  19%  9%  1%  18% | 30  20  18  8  1  17 |
